# An investigation of the associations between stigma, self-compassion, and pain outcomes during treatment based on Acceptance and Commitment Therapy for chronic pain

**DOI:** 10.3389/fpsyg.2024.1322723

**Published:** 2024-02-06

**Authors:** Madeleine Anderson, Lance M. McCracken, Whitney Scott

**Affiliations:** ^1^INPUT Pain Unit, Guy’s and St. Thomas’ Hospital NHS Foundation Trust, London, United Kingdom; ^2^Department of Psychology, Uppsala University, Uppsala, Sweden; ^3^Health Psychology Section, Institute of Psychiatry, Psychology, and Neuroscience, King’s College London, London, United Kingdom

**Keywords:** stigma, self-compassion, psychological flexibility, chronic pain, acceptance and commitment therapy

## Abstract

**Introduction:**

Stigma adversely affects people with chronic pain. The qualities within self-compassion may be particularly useful for buffering the impact of stigma on people with pain. In the context of an Acceptance and Commitment Therapy-based (ACT) treatment for chronic pain, this study investigated the association between changes in stigma and self-compassion and pain outcomes, and the potential moderating role of self-compassion on the association between stigma and pain outcomes.

**Materials and methods:**

Five-hundred and nineteen patients completed standardized self-report questionnaires of stigma, self-compassion, psychological flexibility, pain intensity and interference, work and social adjustment, and depression symptoms at the start of an interdisciplinary ACT-based treatment for chronic pain. The same measures were completed at post-treatment (*n* = 431).

**Results:**

The results indicated that key pain outcomes and self-compassion significantly improved during treatment, but stigma did not. Changes in stigma and self-compassion were significantly negatively correlated and changes in these variables were associated with improvements in treatment outcomes. There were significant main effects of stigma and self-compassion for many of the pre- and post-treatment regression models when psychological flexibility was not controlled for, but self-compassion did not moderate the association between stigma and pain outcomes. Stigma remained significant when psychological flexibility variables were controlled for, while self-compassion did not.

**Discussion:**

The findings add to our conceptual understanding of the inter-relationships between stigma, self-compassion, and psychological flexibility and can contribute to treatment advancements to optimally target these variables.

## Introduction

Definitions of chronic pain widely recognize that it comprises a complex interaction between biological, psychological and social processes (Williams and Craig, 2016; Clauw et al., 2019). Whilst the role of psychological factors has been well researched in people with pain, less research has focused on social factors. Research indicates that supportive and empathetic social interactions promote positive outcomes in people with chronic pain (Cano and Williams, 2010), whilst negative social responses, characterized by a lack of understanding or rejection, are linked to poorer mental and physical health (Kool et al., 2013; Scott et al., 2019; Bean et al., 2022). The absence of a clear medical explanation for many chronic pain conditions can result in patients’ experiences being discredited, which can contribute to stigmatizing or invalidating responses from others (Martel et al., 2012; De Ruddere and Craig, 2016).

Stigma is defined as devaluing or discrediting an individual or group because they possess characteristics that are perceived to be different from societal norms (Goffman, 1963). It can be separated into “enacted” stigma, which is the overt act of poor or unfair treatment toward an individual or group, and “internalised” stigma which describes when the stigmatized individual comes to view negative self-referential attitudes as true (Molina et al., 2013). Importantly, in people with chronic pain, those experiencing greater stigma show poorer psychological wellbeing, and greater disability, pain intensity and depressive symptoms (Waugh et al., 2014; Scott et al., 2019; Bean et al., 2022). Therefore, interventions are needed to target stigma and its impact on pain outcomes (De Ruddere and Craig, 2016). Given the multifaceted nature of stigma, systemic (e.g., more inclusive disability policies), interpersonal (e.g., more validating communication toward people with pain), and individual interventions (e.g., reducing the impact of stigma-related thoughts and feelings) are all necessary to improve the lives of people with pain (De Ruddere and Craig, 2016; Stangl et al., 2019).

A previous observational study explored change in stigma following an interdisciplinary pain management treatment based on acceptance and commitment therapy (ACT) for chronic pain (Scott et al., 2019). Total stigma scores, a summary that included enacted and internalized stigma, did not change, although there was a small reduction in internalized stigma (Scott et al., 2019). The lack of change in stigma is perhaps unsurprising as the intervention did not directly address wider social systems that feed into stigma (Stangl et al., 2019).

Notably, ACT does not aim to directly reduce or change difficult pain-related thoughts and feelings (McCracken, 2023), which may include those related to stigma. Instead, ACT aims to improve mental health and daily physical and social functioning in the presence of pain and related difficulties. Consistent with this, data from randomized-controlled trials indicate that ACT produces larger impacts on depression and disability outcomes than pain intensity (McCracken et al., 2022). Theoretically, ACT targets improved mental health and daily functioning through fostering psychological flexibility, which is the ability to remain aware of and open to difficult experiences moment-by-moment, and to flexibly engage in meaningful activities in the presence of challenges (Kashdan and Rottenberg, 2010; Hayes et al., 2011). A growing body of studies shows that psychological flexibility processes, including pain acceptance, present-moment awareness, and values-based/committed action improve following ACT (McCracken et al., 2022), as do fear-avoidance model constructs and self-efficacy which are targets of traditional cognitive-behavioral therapy (Wicksell et al., 2013; Varallo et al., 2023). Improvements in psychological flexibility processes are associated with improvements in key pain-related outcomes like depression and daily functioning (Feliu-Soler et al., 2018; McCracken, 2023). The magnitude of association between changes in psychological flexibility and pain intensity tend to be inconsistent and smaller than the links between psychological flexibility and the other pain outcomes (McCracken, 2023). Taken together, this suggests that rather than directly changing experiences of stigma, ACT-relevant processes may alter the impact of stigma on key pain outcomes, although research is needed to understand the relationships between these variables.

Self-compassion (SC) shares similar features with psychological flexibility, features that may be particularly important for counteracting the impact of stigma. Self-compassion involves acting with kindness toward the self, mindful awareness, and a sense of common humanity in suffering, rather than self-criticism, overidentifying with challenges, or isolation (Neff, 2003; Germer and Neff, 2013). Self-kindness, mindfulness, and common humanity are conceptually related to the “aware,” “open,” and “engaged” facets of psychological flexibility (Hayes et al., 2011), and research shows that measures of self-compassion and facets of psychological flexibility are partially overlapping (Neff and Tirch, 2013; Carvalho et al., 2019, 2020; Davey et al., 2020; Kılıç et al., 2022). Self-compassion is associated with indices of improved functioning and well-being (Neff et al., 2007; Yang and Mak, 2017), including in people with chronic pain (Edwards et al., 2019; Carvalho et al., 2020; Davey et al., 2020). Given their overlap, ACT-based interventions that aim to improve psychological flexibility may also improve self-compassion (Yadavaia et al., 2014). However, the potential impact of ACT on self-compassion needs to be examined in people with chronic pain.

Research is needed to understand the links between stigma, self-compassion, and chronic pain outcomes. Given the qualities within self-compassion, it is plausible that it can buffer the impact of stigma on chronic pain outcomes. Indeed, outside the pain field, Yang and Mak (2017) found that self-compassion moderates the relationship between self-stigma and subjective wellbeing in a sample of people living with HIV, wherein greater self-compassion reduced the adverse effect of self-stigma on wellbeing. As both HIV and chronic pain populations are at risk of experiencing stigma, it is possible that this moderating relationship may also apply to a chronic pain sample. This needs to be tested.

Therefore, this study investigated the associations between stigma, self-compassion, psychological flexibility, and treatment outcomes during an ACT-based pain management treatment. It was predicted that the treatment would be associated with improvements in psychological flexibility, self-compassion, and key pain outcomes (i.e., pain intensity, and interference, work and social adjustment, and depression), and that any changes in stigma would correlate with changes in these other variables. We also hypothesized that at pre- and post-treatment, higher levels of self-compassion would reduce the strength of associations between stigma and each of the pain outcomes. We examined this moderation hypothesis separately at pre- and post-treatment given previous findings that stigma did not change during an ACT-based treatment for pain (Scott et al., 2019), which would make it difficult to interpret longitudinal moderation analyses.

## Methods

### Participants

All participants (*n* = 519) were adults with chronic pain attending a residential pain management treatment at St Thomas’ Hospital in London. The treatment was interdisciplinary and based on ACT principles aiming to improve quality of life and daily functioning with pain. To determine suitability for the treatment, all participants were assessed by a physiotherapist and psychologist. They were deemed eligible if they were over 18 years old, had chronic pain (≥3 months) significantly impacting their quality of life and functioning, and showed willingness to attend a group-based treatment that was not focused on reducing pain. Exclusion criteria included the inability to manage their own self-care independently, significant ongoing medical procedures or investigations, and psychological conditions restricting their ability to safely participate (e.g., active psychosis, active suicidal intent).

Judgments about participant eligibility and the suitability of the treatment were made following discussion between the assessing psychologist and physiotherapist based on information obtained during a semi-structured assessment interview. Physiotherapists subjectively assessed the impact of pain on patients’ physical activities and observed behavioral indicators of this (e.g., gait, range of motion) during the assessment interview. However, more “objective” indicators of physical functioning were not assessed. This is because standardized “objective” measures of physical functioning (e.g., distance walked in 5 min) emphasize the quantity rather than the quality of movement or whether a particular movement is important to the individual, both of which are more relevant within the ACT model (Guildford et al., 2017).

### Procedure

**Figure 1:**
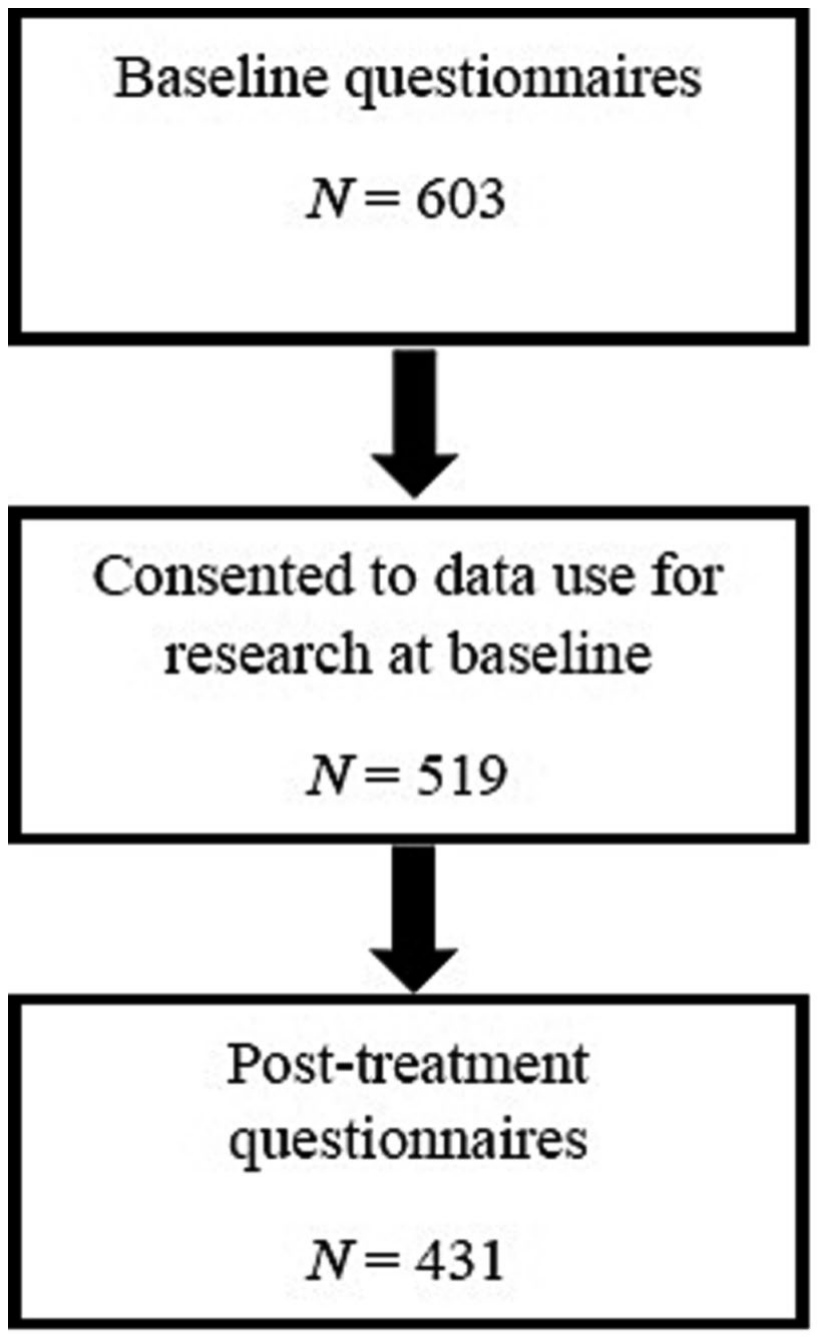
Questionnaire completion across treatment timepoints. Data missing on individual questionnaires at baseline: SCS-SF, 36; SSCI-8, 26; Pain intensity, 4; BPI-IS, 1; WSAS, 2; PHQ-9, 10; CAQ-8, 20; SEQ, 85; CFQ-7, 15; CPAQ-8, 16. Data missing on individual questionnaires at post-treatment: SCS-SF, 36; SSCI-8, 32; Pain intensity, 17; BPI-IS, 20; WSAS, 21; PHQ-9, 21; CAQ-8, 26; SEQ, 55; CFQ-7, 21; CPAQ-8, 24.

Participants were consecutively sampled between January 2018 and October 2019 and asked to complete a standardized set of paper-based self-report assessment measures on the first day of treatment. Alongside those summarized below, measures collected demographic data such as age, ethnicity, living status, work status, and pain characteristics. At the end of treatment, participants completed the same measures again (excluding demographics) to provide post-treatment data. Informed consent was obtained from participants to use their data for research purposes and ethical approval was granted from the South Central Oxford NHS research committee (17/SC/0537). See Figure 1 for the number of participants accrued across the data collection process. Portions of the baseline data have been reported in a previous paper (Davey et al., 2020). However, that paper did not report any of the post-treatment data.

Participants completed an intensive group-based residential treatment for three-weeks (four days per week). The treatment was based on ACT (Hayes et al., 2011; McCracken and Morley, 2014; McCracken et al., 2022). Therefore, it aimed to increase psychological flexibility to support engagement in meaningful life activities in the presence of pain, and it did not focus on reducing or controlling pain. Experiential exercises, metaphors, mindfulness practices, values clarification and values-based goal-setting were used across the interdisciplinary team, which included psychologists, physiotherapists, occupational therapists, and nurses. Given the size of the service and that more than one treatment group often ran in parallel, a few different teams of multidisciplinary clinicians (reflecting each of the disciplines) delivered the treatment across the different groups. Fidelity to the ACT model was supported through regular reflective team meetings and clinical development sessions attended by all the multidisciplinary clinicians who delivered treatment across the different groups. Although this was not a compassion-focused treatment, clinicians within the service draw on concepts related to self-compassion as relevant in their work with the psychological flexibility processes within ACT.

### Assessment measures

#### Self-compassion

##### Self-Compassion Scale – Short Form (SCS-SF)

The 12-item SCS-SF (Raes et al., 2011) is a truncated version of the Self-Compassion Scale (Neff, 2003), measuring the same six key components (self-kindness, self-judgment, common humanity, isolation, mindfulness and over-identification) using a 5-point scale ranging from 1 (Very rarely true) to 5 (Almost always true). An overall score is calculated by reverse scoring self-judgment, isolation, and over-identification items, and then calculating the mean of all items, wherein higher scores indicate greater self-compassion. The SCS-SF has been utilized in chronic pain samples (Carvalho et al., 2018; Davey et al., 2020) and has been validated as a reliable alternative to the full-length SCS (Raes et al., 2011).

#### Stigma

##### Stigma Scale for Chronic Illnesses (SSCI-8)

Across eight items, the SSCI-8 (Molina et al., 2013) assesses how often individuals experience internalized (e.g., “I felt embarrassed about my illness”) and enacted (e.g., “because of my illness, some people avoided me”) stigma, on a scale from 1 (Never) to 5 (Always). Consistent with previous research showing that the SSCI-8 has a unidimensional factor structure (Molina et al., 2013; Scott et al., 2019), all items were summed to produce a total score, with higher scores representing greater stigma. Previous research has supported the validity and reliability of this measure for use in clinical populations, including people with chronic pain (Molina et al., 2013; Scott et al., 2019).

#### Outcome variables

##### Pain intensity

This measure asks participants to rate how intense their pain had been over the past week on average, on a scale from 0 (no pain) to 10 (pain as bad as you can imagine).

##### Brief Pain Inventory – Interference Scale (BPI-IS)

The impact of pain on functioning was assessed using the BPI-IS, which asks participants to rate how much pain has interfered with various daily activities during the past week (Cleeland and Ryan, 1994). The seven activities (general activity, mood, walking ability, normal work, relations with other people, sleep, enjoyment of life) are rated on a scale from 0 (does not interfere) to 10 (completely interferes). The mean of the seven items is calculated to produce an overall interference score, where higher scores represent greater pain-related interference. This is a commonly used and well-validated measure of pain interference (Keller et al., 2004; Atkinson et al., 2011). The BPI-IS was included in addition to the Work and Social Adjustment Scale (WSAS; see below) due to the different facets of functioning captured by both measures; the WSAS provides a more in-depth insight into social and leisure activities whereas the BPI-IS is able to assess functioning across a wider spread of daily activities.

##### Work and Social Adjustment Scale (WSAS)

The WSAS was included to measure the level of functional impairment across aspects of life, including relationships, social and private leisure activities, home management and ability to work (Mundt et al., 2002). The five items are rated on a 9-point scale ranging from 0 (no impairment) to 8 (very severe impairment) and summed to produce a total score, with higher scores indicating a more severe level of impairment. The WSAS is considered a valid and reliable measure of functional impairment and has demonstrated good internal and construct validity within clinical settings (Mundt et al., 2002; Cella et al., 2011).

##### Patient Health Questionnaire (PHQ-9)

The severity of depression symptoms was assessed using the PHQ-9 (Kroenke et al., 2001). On this measure, participants are asked how often they have been bothered by a variety of symptoms over the last two weeks, and instructed to rate each item on a 4-point scale ranging 0 (not at all) to 3 (nearly every day). Summing the nine items produces a total score, wherein a higher value indicates a higher level of depression symptoms. The PHQ-9 has demonstrated construct and criterion validity, and has been deemed a valid and reliable measure of depression symptoms (Kroenke et al., 2001; Kroenke and Spitzer, 2002; Gilbody et al., 2007).

#### Psychological flexibility/inflexibility measures

##### Chronic Pain Acceptance Questionnaire (CPAQ-8)

Pain-related acceptance was measured using the eight-item CPAQ-8 (Fish et al., 2010). Items capture willingness to experience pain rather than struggling to control pain (pain willingness), and the extent to which an individual engages with meaningful life activities in the presence of pain (activity engagement) (Fish et al., 2013). All items are rated from 0 (Never true) to 6 (Always true), and higher total scores indicated increased pain-related acceptance. This measure exhibits good convergent validity with the original 20-item CPAQ, and is considered to be valid and reliable (Fish et al., 2010; Baranoff et al., 2014).

##### Cognitive Fusion Questionnaire (CFQ-7)

The CFQ-7 is a seven-item measure assessing the extent to which an individual is unable to separate themselves and their behavior from their thoughts (Gillanders et al., 2014). Participants rate statements from 1 (Never true) to 7 (Always true), with a higher total score reflecting greater cognitive fusion and therefore greater psychological *inflexibility*. The CFQ-7 has shown good internal consistency and is considered a reliable measure of cognitive fusion among people with chronic pain (Gillanders et al., 2014; McCracken et al., 2014).

##### Committed Action Questionnaire (CAQ-8)

On the CAQ-8, participants are presented with eight statements concerning valued or goal-directed action, and asked to rate how much these apply to themselves on a 7-point scale ranging from 0 (never true) to 6 (always true) (McCracken et al., 2015). A total score is calculated by summing all items, with items 5 to 8 reverse scored. A higher score indicates greater committed action and therefore greater psychological flexibility. As a shortened version of the original 18-item CAQ, the CAQ-8 has demonstrated good construct validity and internal consistency (McCracken et al., 2015; Åkerblom et al., 2016; Wong et al., 2016).

##### Self-Experiences Questionnaire (SEQ)

This fifteen-item scale is a measure of self-as-context, another core psychological flexibility process (Yu et al., 2016). Items are designed to capture the extent to which individuals take an observational perspective of their thoughts and feelings (e.g., “I can observe experiences in my body and mind as events that come and go”) and their awareness of thoughts and feelings as distinct from the self (e.g., “I have thoughts and feelings but am not defined as just my thoughts and feelings”). This measure was designed and validated within chronic pain samples, and has shown good reliability and adequate internal consistency (Yu et al., 2016, 2017). All items are summed to produce a total score, wherein higher scores suggest a stronger self-as-context.

### Statistical analysis

Statistical analyses were conducted using SPSS version 27. Descriptive statistics were calculated for baseline demographic variables and for study variables at pre- and post-treatment. Using Jamovi Cloud, paired-sample *t*-tests were computed for all study variables to investigate the magnitude of change from pre- to post-treatment. For effect sizes (*d*) Cohen’s thresholds were used, wherein *d* = 0.2 indicates a small effect size, *d* = 0.5 medium, and *d* = 0.8 large (Cohen, 2013). Residualized change scores were computed for all process and outcome variables, and these scores were used to compute bivariate Pearson’s correlations to investigate the associations between changes on these variables.

Hierarchical regression analyses were conducted to test the moderating effect of self-compassion on stigma at pre- and post-treatment for the four outcomes of interest; pain intensity, pain interference, work and social impairment, and depression. As mentioned, we examined the moderating role of self-compassion separately at pre- and post-treatment as stigma was not expected to change significantly (Scott et al., 2019), limiting the interpretability of longitudinal moderation analyses. Pain intensity was entered in step one (except for the model with pain intensity as the outcome), followed by demographic variables (ethnicity, work status, gender) at step two as covariates. Demographic variables entered at step two were selected due to having significant correlations with each outcome variable in preliminary analyses. Due to a high volume of missing data for age (*n* = 106), this variable was excluded from the regression models to maximize power. At step three, self-compassion and stigma were entered, and finally the stigma and self-compassion interaction term at step four. The interaction term was created using centered variables to reduce multicollinearity.

Additional regression analyses were run which included psychological flexibility/inflexibility variables to investigate what effect these may have on the unique contribution of self-compassion. Model structure was identical to that outline above, although the psychological flexibility variables were entered at step four, and therefore the stigma/self-compassion interaction term was entered at step five. For the regression analyses, a Bonferroni correction was applied whereby the alpha level of *p* < 0.05 was divided by 8, which was the number of regression models (corrected alpha, *p* < 0.006).

## Results

### Sample characteristics

Participants were aged between 19 and 85 years (*M* = 48.18, SD = 13.04) and the majority (78.4%) were women. The mean duration of pain was 13 years (SD = 11.21). Further demographic details of the sample are shown in Table 1. Participants who completed post-treatment questionnaires showed significantly less baseline cognitive fusion (*M* = 32.13, SD = 11.30) than those who did not complete these questionnaires [(*M* = 34.77, SD = 10.40), *t*(502) = 1.998, *p* < 0.05]. For all other baseline demographic and study variables, there were no significant differences between people who did and did not provide post-treatment data.

**Table 1:** Demographic characteristics of the sample.

	Mean ± SD or *N* (%)	
Age, y (missing = 106)	48.18 ±	13.04	Pain duration, y (missing = 77)	13.61 ±	11.21
Gender		
Men	109	(21.0)
Women	407	(78.4)
Missing	3	(0.6)
Ethnicity		
Asian	39	(7.5)
Black	62	(11.9)
Mixed	22	(4.2)
Other	11	(2.1)
White	373	(71.9)
Missing	12	(2.3)
Living status		
With partner and child/children	128	(24.7)
Alone	120	(23.1)
With partner or spouse	117	(22.5)
With child/children	78	(15.0)
With other relatives	52	(10.0)
With friends or flatmates	13	(2.5)
Missing	11	(2.1)
Work status		
Unemployed because of pain	269	(51.8)
Employed full time	48	(9.2)
Employed part-time due to pain	70	(13.5)
Retired	58	(11.2)
Unemployed for other reason	15	(2.9)
Homemaker	12	(2.3)
Employed part-time for other reason	12	(2.3)
Unpaid volunteer	7	(1.3)
Carer	3	(0.6)
Student	5	(1.0)
In other training	3	(0.6)
Missing	17	(3.3)
Primary pain location		
Lower back, lumbar spine, sacrum and coccyx	198	(38.2)
Generalized	89	(17.1)
Lower limbs	58	(11.2)
Neck region	32	(6.2)
Upper shoulder or upper limbs	29	(5.6)
Head, face or mouth	22	(4.2)
Abdominal region	20	(3.9)
Pelvic region	10	(1.9)
Chest region	6	(1.2)
Anal or genital region	2	(0.4)
Missing	53	10.2

### Pre- to post-treatment change on study variables

Descriptive statistics and paired-sample *t*-tests for variables at pre- versus post-treatment are shown in Table 2. A significant difference between the two timepoints was found for most variables showing an improvement after treatment, with the exception of stigma, committed action, and cognitive fusion. Of the variables that showed a statistically significant improvement, the magnitude of effects ranged from small for self-compassion and self-as-context (both *d* = 0.21) to large for pain interference (*d* = 0.89) and depression (*d* = 0.88).

**Table 2:** Descriptive statistics and paired-sample *t*-test results for variables at pre- versus post-treatment.

	Pre-treatment		Post-treatment		*t*	*df*	*p*	*d*
	*M*	*SD*	*M*	*SD*				
Self-compassion (SCS-SF)	2.60	0.75	2.74	0.75	−4.14	375	<0.001	0.21
Stigma (SSCI-8)	23.27	7.53	22.83	7.69	1.53	386	0.128	0.08
Pain intensity	7.79	1.51	7.02	1.79	9.93	409	<0.001	0.49
Pain interference (BPI-IS)	7.80	1.57	6.40	1.90	17.98	409	<0.001	0.89
Work and social impairment (WSAS)	32.48	6.18	29.27	7.77	9.59	407	<0.001	0.47
Depression (PHQ-9)	17.93	5.51	12.99	6.43	17.74	401	<0.001	0.88
Committed action (CAQ-8)	25.69	8.28	26.27	8.27	−1.59	392	0.115	0.08
Self-as-context (SEQ)	46.64	16.55	49.83	15.77	−3.90	333	<0.001	0.21
Cognitive fusion (CFQ-7)	32.18	11.31	31.82	11.08	0.86	400	0.388	0.04
Pain acceptance (CPAQ-8)	16.65	8.02	20.15	8.38	−8.45	397	<0.001	0.42

Bonferroni-corrected alpha for pre- to post-treatment change analyses (0.05/10) was *p* < 0.005. SCS-SF, Self-Compassion Scale – Short form; SSCI-8, Stigma Scale for Chronic Illnesses – eight item version; BPI-IS, Brief Pain Inventory – Interference Scale; WSAS, Work and Social Adjustment Scale; PHQ-9, Patient Health Questionnaire; CAQ, Committed Action Questionnaire – eight item version; SEQ, Self-Experiences Questionnaire; CFQ, Cognitive Fusion Questionnaire; CPAQ, Chronic Pain Acceptance Questionnaire – eight item version. Differences in df across variables is due to missing data on individual variables, as summarized in the note at Figure 1.

### Bivariate associations between changes on study variables

Pearson’s correlation coefficients between change scores on all variables from pre- to post-treatment are displayed in Table 3. Changes in stigma and self-compassion were significantly negatively correlated (medium effect) and changes in these variables were associated with improvements in each of the treatment outcomes (small to medium effects).

**Table 3:** Correlations among pre- and post-treatment change scores for study variables.

	1	2	3	4	5	6	7	8	9	10
Self-compassion	-									
Stigma	−0.406**	-								
Pain intensity	−0.135**	0.128*	-							
Pain interference	−0.351**	0.386**	0.549**	-						
Work and social impairment	−0.372**	0.329**	0.357**	0.621**	-					
Depression	−0.354**	0.364**	0.382**	0.555**	0.527**	-				
Committed action	0.466**	−0.287**	−0.094	−0.219**	−0.302**	−0.333**	-			
Self-as-context	0.506**	−0.238**	−0.103	−0.210**	−0.241**	−0.191**	0.401**	-		
Cognitive fusion	−0.500**	0.296**	0.116*	0.294**	0.379**	0.351**	−0.479**	−0.326**	-	
Pain acceptance	0.526**	−0.311**	−0.170**	−0.372**	−0.505**	−0.363**	0.495**	0.438**	−0.447**	-

*p < 0.05; **p < 0.01.

### Moderation analyses: simpler models

At baseline, work status was significantly negatively correlated with pain intensity (*r*_pb_ = −0.22, *p* < 0.001), pain interference (*r*_pb_ = −0.27, *p* < 0.001), depression (*r*_pb_ = −0.18, *p* < 0.001), and work and social impairment (*r*_pb_ = −0.31, *p* < 0.001). Ethnicity (dichotomized as ethnically minoritized = 0, and white = 1) was significantly negatively correlated with pain intensity (*r*_pb_ = −0.17, *p* < 0.001) and pain interference (*r*_pb_ = −0.13, *p* = 0.005). Gender showed no significant associations with any outcome variables. As a result, only ethnicity and work status were included in regression models.

Bivariate associations showed age was significantly, but only weakly, correlated with stigma at baseline (*r* = −0.20, *p* < 0.001) and post-treatment (*r* = −0.22, *p* < 0.001) and very weakly correlated with baseline self-compassion (*r* = 0.19, *p* < 0.001). However, of the four pain outcome variables, only baseline pain intensity (*r* = 0.15, *p* = 0.002) showed a very weak significant correlation with age. Given the lack of associations between age and the pain outcomes and the large amount of missing data on age (as previously mentioned), age was not included as a covariate in the models.

No significant interaction effects were found at the specified Bonferroni correction level (*p* < 0.006) for any of the outcome variables in the simpler models. For baseline depression, there were significant main effects of self-compassion (*β* = −0.18, *p* < 0.001) and stigma (*β* = 0.22, *p* < 0.001). At post-treatment, there were also significant main effects of self-compassion (*β* = −0.14, *p* < 0.001) and stigma (*β* = 0.27, *p* < 0.001) on depression. A significant main effect of self-compassion was observed at post-treatment for pain interference (*β* = −0.03, *p* = 0.003) and work and social impairment (*β* = −0.15, *p* < 0.001). There was a significant main effect of stigma for pain interference at baseline (*β* = 0.05, *p* < 0.001) and post-treatment (*β* = 0.05, *p* < 0.001), and for work and social impairment at baseline (*β* = 0.25, *p* < 0.001) and post-treatment (*β* = 0.26, *p* < 0.001), but only at post-treatment for pain intensity (*β* = 0.05, *p* < 0.001).

### Models including psychological flexibility/inflexibility variables

Given the conceptual similarity between self-compassion and psychological flexibility constructs, further regression analyses were conducted to determine if the significant unique contribution of self-compassion remained in the presence of psychological flexibility/inflexibility variables (entered at step 4). These full regression analyses are shown in Tables 4–7. After the addition of psychological flexibility/inflexibility variables, self-compassion no longer made a significant unique contribution to any of the outcome variables at pre- or post-treatment. Of the psychological flexibility/inflexibility variables, pain acceptance was a significant unique predictor in all models, except for pain intensity (both time points) and pre-treatment depression. Cognitive fusion was a significant unique predictor of pre- and post-treatment depression. Stigma remained a significant unique predictor for all outcomes at pre- and post-treatment, except for pre-treatment pain intensity. There were no significant interaction effects.

**Table 4:** Hierarchical regression analyses examining the association between stigma, self-compassion, and pain intensity.

Step	Independent variable	Pre-treatment						Post-treatment					
		*F* change	*df*	*p*	Adjusted *R^2^*	*β*	*p*	*F* change	*df*	*p*	Adjusted *R^2^*	*β*	*p*
Dependent variable: Pain intensity		
1	Work status (no/yes)	16.81	(2, 385)	<0.001	0.08	−0.69	<0.001	11.69	(2, 341)	<0.001	0.06	−0.66	0.001	Ethnicity^a^					−0.54	0.002					−0.22	0.301
2	Self-compassion (SCS-SF)	2.07	(2, 383)	0.128	0.08	0.01	0.586	11.44	(2, 339)	<0.001	0.11	0.01	0.474	Stigma (SSCI-8)					0.02	0.067					0.05	0.002
3	Committed action (CAQ)	0.41	(4, 379)	0.801	0.08	0.01	0.240	1.95	(4, 335)	0.102	0.12	0.00	0.948	Cognitive fusion (CFQ)					0.01	0.514					0.01	0.717	Pain acceptance (CPAQ)					−0.01	0.627					−0.04	0.016	Self-as-context (SEQ)					0.00	0.996					0.00	1.00
4	Stigma x Self-compassion	0.08	(1, 378)	0.774	0.07	0.00	0.774	0.26	(1, 334)	0.612	0.12	−0.00	0.612

*β* coefficients and associated *p* values are from the final regression model. Bonferroni-corrected alpha for regression analyses (0.05/8) was *p* < 0.006. ^a^Ethnicity coded as: Ethnically minoritized = 0; White = 1. SCS-SF, Self-Compassion Scale – Short form; SSCI-8, Stigma Scale for Chronic Illnesses – eight item version; CAQ, Committed Action Questionnaire– eight item version; CFQ, Cognitive Fusion Questionnaire; CPAQ, Chronic Pain Acceptance Questionnaire – eight item version; SEQ, Self-Experiences Questionnaire.

**Table 5:** Hierarchical regression analyses examining the association between stigma, self-compassion, and pain interference.

Step	Independent variable	Pre-treatment						Post-treatment					
		*F* change	*df*	*p*	Adjusted *R^2^*	*β*	*p*	*F* change	*df*	*p*	Adjusted *R^2^*	*β*	*p*
Dependent variable: pain interference (BPI-IS)													
1	Pain intensity	140.14	(1, 386)	<0.001	0.26	0.48	<0.001	251.76	(1, 341)	<0.001	0.42	0.54	<0.001
2	Work status (no/yes)	5.94	(2, 384)	0.003	0.28	−0.29	0.034	6.71	(2, 339)	0.001	0.44	−0.30	0.040
	Ethnicity^a^					−0.06	0.686					−0.21	0.181
3	Self-compassion (SCS-SF)	23.00	(2, 382)	<0.001	0.36	−0.01	0.344	23.47	(2, 337)	<0.001	0.51	−0.00	0.796
	Stigma (SSCI-8)					0.05	<0.001					0.04	<0.001
4	Committed action (CAQ)	6.48	(4, 378)	<0.001	0.39	−0.01	0.367	11.38	(4, 333)	<0.001	0.56	0.02	0.081
	Cognitive fusion (CFQ)					−0.01	0.433					0.00	0.991
	Pain acceptance (CPAQ)					−0.04	<0.001					−0.07	<0.001
	Self-as-context (SEQ)					−0.00	0.401					−0.00	0.596
5	Stigma x Self-compassion	0.88	(1, 377)	0.348	0.39	−0.00	0.348	1.19	(1, 332)	0.277	0.56	−0.00	0.277

*β* coefficients and associated *p* values are from the final regression model. Bonferroni-corrected alpha for regression analyses (0.05/8) was *p* < 0.006. ^a^Ethnicity coded as: Ethnically minoritized = 0; White = 1. BPI-IS, Brief Pain Inventory – Interference Scale; SCS-SF, Self-Compassion Scale – Short form; SSCI-8, Stigma Scale for Chronic Illnesses – eight item version; CAQ, Committed Action Questionnaire; CFQ, Cognitive Fusion Questionnaire; CPAQ, Chronic Pain Acceptance Questionnaire – eight item version; SEQ, Self-Experiences Questionnaire.

**Table 6:** Hierarchical regression analyses examining the association between stigma, self-compassion, and work and social impairment.

Step	Independent variable	Pre-treatment						Post-treatment					
		*F* change	*df*	*p*	Adjusted *R^2^*	*β*	*p*	*F* change	*df*	*p*	Adjusted *R^2^*	*β*	*p*
Dependent variable: work and social impairment (WSAS)													
1	Pain intensity	54.45	(1, 393)	<0.001	0.12	1.12	<0.001	131.25	(1, 345)	<0.001	0.27	1.42	<0.001
2	Work status (no/yes)	28.15	(1, 392)	<0.001	0.18	−2.40	<0.001	31.21	(2, 344)	<0.001	0.33	−2.85	<0.001
3	Self-compassion (SCS-SF)	24.99	(2, 390)	<0.001	0.27	0.08	0.064	31.22	(2, 342)	<0.001	0.43	−0.01	0.816
	Stigma (SSCI-8)					0.23	<0.001					0.19	<0.001
4	Committed action (CAQ)	9.37	(4, 386)	<0.001	0.33	−0.03	0.490	18.45	(4, 338)	<0.001	0.53	0.07	0.146
	Cognitive fusion (CFQ)					0.00	0.989					0.02	0.599
	Pain acceptance (CPAQ)					−0.22	<0.001					−0.39	<0.001
	Self-as-context (SEQ)					−0.01	0.735					−0.00	0.936
5	Stigma x Self-compassion	0.00	(1, 385)	0.972	0.32	−0.00	0.972	1.60	(1, 337)	0.206	0.53	0.01	0.206

*β* coefficients and associated *p* values are from the final regression model. Bonferroni-corrected alpha for regression analyses (0.05/8) was *p* < 0.006. WSAS, Work and Social Adjustment Scale; SCS-SF, Self-Compassion Scale – Short form; SSCI-8, Stigma Scale for Chronic Illnesses – eight item version; CAQ, Committed Action Questionnaire – eight item version; CFQ, Cognitive Fusion Questionnaire; CPAQ, Chronic Pain Acceptance Questionnaire – eight item version; SEQ, Self-Experiences Questionnaire.

**Table 7:** Hierarchical regression analyses examining the association between stigma, self-compassion, and depression.

Step	Independent variable	Pre-treatment						Post-treatment					
		*F* change	*df*	*p*	Adjusted *R^2^*	*β*	*p*	*F* change	*df*	*p*	Adjusted *R^2^*	*β*	*p*
Dependent variable: depression (PHQ-9)													
1	Pain intensity	30.73	(1, 386)	<0.001	0.07	0.78	<0.001	106.54	(1, 344)	<0.001	0.23	1.22	<0.001
2	Work status (no/yes)	9.74	(1, 385)	0.002	0.09	−0.91	0.073	1.19	(1, 343)	0.277	0.24	0.33	0.554
3	Self-compassion (SCS-SF)	58.65	(2, 383)	<0.001	0.30	−0.04	0.255	46.14	(2, 341)	<0.001	0.39	0.02	0.643	Stigma (SSCI-8)					0.16	<0.001					0.17	<0.001
4	Committed action (CAQ)	7.98	(4, 379)	<0.001	0.35	−0.04	0.243	9.33	(4, 337)	<0.001	0.45	0.00	0.930	Cognitive fusion (CFQ)					0.12	<0.001					0.14	<0.001	Pain acceptance (CPAQ)					−0.04	0.174					−0.15	<0.001	Self-as-context (SEQ)					−0.01	0.522					−0.01	0.828
5	Stigma x Self-compassion	1.16	(1, 378)	0.283	0.35	0.00	0.283	2.10	(1, 336)	0.148	0.45	−0.01	0.148

*β* coefficients and associated *p* values are from the final regression model. Bonferroni-corrected alpha for regression analyses (0.05/8) was *p* < 0.006. PHQ-9, Patient Health Questionnaire Depression Module; SCS-SF, Self-Compassion Scale – Short form; SSCI-8, Stigma Scale for Chronic Illnesses – eight item version; CAQ, Committed Action Questionnaire; CFQ, Cognitive Fusion Questionnaire; CPAQ, Chronic Pain Acceptance Questionnaire – eight item version; SEQ, Self-Experiences Questionnaire.

## Discussion

This study examined the associations between stigma, self-compassion, psychological flexibility, and chronic pain outcomes. The results indicated that key pain outcomes, self-compassion, pain acceptance and self-as-context significantly improved during an ACT-based treatment, but stigma, cognitive fusion, and committed action did not. Changes in stigma and self-compassion were significantly negatively correlated and changes in these variables were associated with improvements in each of the treatment outcomes. There were significant main effects of stigma and self-compassion for many of the simpler pre- and post-treatment regression models. However, the moderation hypothesis was not supported. The role of stigma remained significant when psychological flexibility variables were included in the models, while self-compassion did not. Taken together, these results add to our conceptual understanding of the inter-relationships between stigma, self-compassion, and psychological flexibility and can contribute to treatment advancements to optimally target these variables.

There has been growing interest in the links between self-compassion and psychological flexibility (Moreno, 2023). In particular, the mindfulness elements of self-compassion and psychological flexibility have clear conceptual overlap (Neff and Tirch, 2013). The current study provides further evidence for the association between these variables, with moderate to large bivariate correlations for change scores on these variables, which is consistent with meta-analysis findings (Moreno, 2023). When psychological flexibility processes were controlled for, self-compassion was not significant in any of the regression models in the current study. In a previous study using only the pre-treatment data from the current cohort, self-compassion uniquely predicted depression when controlling for pain acceptance, self-as-context, and committed action (Davey et al., 2020). However, that model did not include cognitive fusion or stigma (Davey et al., 2020). Therefore, the unique predictive utility of self-compassion may be reduced when additional psychological (in)flexibility processes and aspects of the social environment are controlled for. A longitudinal study in people with diabetes also found that only psychological flexibility was a significant unique predictor of distress outcomes; self-compassion was not significant when psychological flexibility was included in the model (Kılıç et al., 2022). Taken together, a growing body of research suggests that psychological flexibility and self-compassion are overlapping but partially distinct variables.

Given the overlap between psychological flexibility and self-compassion, it is perhaps unsurprising that self-compassion improved significantly in the current ACT-based treatment. However, it is notable that, while significant, the magnitude of improvement was small. For comparison, a recent study reporting outcomes from an 8-week pain management treatment based on compassion-focused therapy reported a significant improvement in self-compassion with a large effect (Malpus et al., 2023). That study specifically selected treatment participants who displayed a pattern of “striving,” such that they tended to push through activities or use activities to distract from pain resulting in a “boom and bust” pattern (Malpus et al., 2023). Additionally, there can be many barriers to engaging in self-compassion, such as fears that doing so may lower personal standards or lead to failure, and beliefs that one might not be deserving of self-compassion (Gilbert et al., 2011; Carvalho et al., 2021; Biskas et al., 2022). Theoretically, the functions of such feelings and beliefs could be targeted within an ACT framework, but this may require a longer timeframe than the current three-week treatment, and more specific targeting in the methods used, to support people to respond to differently. Where self-compassion appears to be particularly relevant for someone living with pain, there may be added benefit of supplementing ACT with more in-depth methods from CFT.

Consistent with previous findings, stigma scores did not significantly improve during the current ACT-based treatment for pain (Scott et al., 2019). Nonetheless, bivariate correlations showed a significant moderate negative correlation between change in stigma and self-compassion. In addition to mindful awareness, the behaviors of self-kindness and common humanity in suffering within self-compassion seem particularly relevant for buffering the impact of stigma on pain outcomes. However, the moderation hypothesis was not supported. Thus, the current data suggest that while improvements in self-compassion are associated with improvements in stigma, self-compassion may not reduce the strength of the association between stigma and pain outcomes. This is inconsistent with a previous study which found that self-compassion moderated the association between self-stigma and life satisfaction in people living with HIV (Yang and Mak, 2017). Differences in the nature of the sample, and measurement of stigma and key outcomes in the current study may account for different pattern of moderation results observed here. Additionally, intensive longitudinal data may be needed to capture the dynamic temporal relationships between stigma, self-compassion, and pain outcomes to better understand potential buffering impacts. Therefore, future research might benefit from using ecological momentary assessment methods to capture a more fine-grained picture of how these variables inter-relate in daily life (Van Alboom et al., 2021).

We did not examine moderation of the stigma-pain outcome relationships by psychological flexibility, in part, to reduce type one error given the number of psychological flexibility and outcome measures within this study. A recent study suggests that general psychological acceptance moderates the association between personal stigma and resilience in people living with HIV (Aliche et al., 2022). However, in that study only the Acceptance and Action Questionnaire (AAQ-II) was used as a measure of psychological flexibility. Significant concerns about the validity of the AAQ-II have been raised (Tyndall et al., 2019), and it does not capture all facets of psychological flexibility. To move forward and reduce the possibility of type one error in testing moderation by multiple psychological flexibility processes, future research could use a single comprehensive measure of psychological flexibility. Such comprehensive measures are now available and growing evidence supports their psychometric properties (Francis et al., 2016; Rolffs et al., 2018).

Stigma had a significant main effect in all but one of the regression models that included self-compassion and psychological flexibility processes. These results highlight that, in addition to supporting people to respond to experiences of stigma with self-compassion and flexibility, interventions also need to target wider aspects of the social environment that underlie vulnerability to stigma. This should include efforts to change negative cultural attitudes toward people in pain, eliminate stigmatizing policies, and improve daily interpersonal interactions such as with clinicians, employers, and other people in important professional or personal relationships (Stangl et al., 2019).

Several limitations of the present study must be considered. First and foremost, this study did not use an experimental design. Therefore, causal statements about the impact of treatment or the nature of inter-relationships among variables cannot be made. It is plausible, for example, that greater levels of disability and depression associated with pain contribute to greater levels of stigma. However, research indicates that discrimination experiences, which share overlaps with stigma, predict depression in people with pain over a six-year period, even after controlling for depression and a number of other demographic factors at baseline (Scott et al., 2022). Nonetheless, more intensive longitudinal and experimental designs are needed to make causal inferences about the impact of treatment and the relationships among the studied variables. Single-case experimental designs (SCEDs) may be particularly well suited to efficiently and rigorously advance work in this area (Chisari et al., 2022). For example, SCEDs could be used to explore potential additive effects of compassion-focused strategies to an ACT treatment, and/or whether different individuals show a different pattern of responses to ACT/CFT strategies, which is likely (Sanford et al., 2022). The analysis of group-level data in this study limited our ability to identify individuals who did experience a meaningful change on stigma and/or self-compassion and the nature of how these changes might inter-relate with key pain outcomes within individuals. The frequency of assessments within SCEDs would enable greater understanding of how psychological flexibility, self-compassion, stigma and key pain outcomes relate over time within and between individuals.

The data were collected in the context of a single specialty pain service and therefore it is unclear whether the results may generalize for people living with pain presenting in other contexts. Approximately 25% of the sample was from an ethnically minoritized group. While this is higher than the percentage of ethnically minoritized individuals in England and Wales (18%) [Office for National Statistics (ONS), 2022], this is lower than the percentage specifically in London (46%) where the service is based, although it accepts national referrals. Therefore, the current sample may underrepresent ethnically minoritized groups and, as such, risks perpetuating health inequities (Hood et al., 2022). Finally, there was a large amount of missing data on age which limited our ability to examine age as a covariate within the multiple regression models investigated here.

In conclusion, the current data provide evidence that self-compassion changes significantly within an ACT-based treatment for people with chronic pain, although these changes are small and could be further improved. Changes in stigma and self-compassion were significantly negatively correlated and associated with improvements in treatment outcomes. The data provide further support that self-compassion and psychological flexibility are partially overlapping constructs. Self-compassion may not uniquely contribute to pain outcomes when psychological flexibility is controlled for. Stigma was uniquely associated with pain outcomes at pre- and post-treatment when self-compassion and psychological flexibility were in the models. Self-compassion did not buffer the impact of stigma on key pain outcomes. Alongside individual-level ACT and compassion-focused interventions, systems-level interventions are needed to target the social environment that contributes to stigma.

## Data availability statement

The raw data supporting the conclusions of this article will be made available by the authors upon reasonable request to the corresponding author, without undue reservation.

## Ethics statement

This study involving humans was approved by South Central Oxford NHS research committee (17/SC/0537). This study was conducted in accordance with the local legislation and institutional requirements. The participants provided their written informed consent to participate in this study.

## Author contributions

MA: Conceptualization, Formal analysis, Writing – original draft, Writing – review & editing. LM: Conceptualization, Data curation, Methodology, Writing – review & editing. WS: Conceptualization, Data curation, Methodology, Writing – original draft, Writing – review & editing.
